# Community Wise—effects and participant perceptions of a community- based -positive health intervention for older inhabitants of low SES neighbourhoods: a mixed-methods approach

**DOI:** 10.1186/s12889-023-16148-y

**Published:** 2023-06-27

**Authors:** Feline Platzer, Nardi Steverink, Marieke Haan, Jiska Vorstman, Mathieu de Greef, Martine Goedendorp

**Affiliations:** 1 Department of Health Psychology, University Medical Center Groningen, PO Box 30.001, Hanzeplein 1 9700 RB, Groningen, Netherlands; 2 Faculty of Behavioural and Social Sciences, University of Groningen, Groningen, the Netherlands; 3 Department of Health Studies, Hanze University of Applied Science, Groningen, the Netherlands

**Keywords:** Community-based, health-promotion interventions, Positive health, low socioeconomic status, Older adults, Mixed methods

## Abstract

**Background:**

The Community Wise (CW) intervention applies a community-based approach to improve the physical fitness, self-management ability, loneliness, social cohesion, and well-being of older adults living in neighbourhoods characterized by lower socioeconomic status (SES).

**Methods:**

Participants (*N* = 108) were recruited using several strategies, including door-to-door visits and community key peers. The study was based on a pre-test/post-test design. Outcomes were assessed through mixed methods using questionnaires, performance tests, semi-structured interviews, and focus-group sessions.

**Results:**

Results showed significant improvements on aerobic endurance and shoulder flexibility, but no significant improvements on self-management ability, social cohesion, loneliness, or well-being. Qualitative data analysis did indicate that participants experienced improvements on social connectedness with members of the group, as well as on self-management ability.

**Conclusion:**

The results of the intervention seem to depend on programme fidelity and method of assessment. Adapting the intervention and including more older adults with poor health status could lead to better outcomes in the future. This results of this study should be interpreted in light of the complexity and methodological challenges of conducting a community-based health-promotion intervention for this target group.

**Trail registration:**

Retrospective registration.

**Supplementary Information:**

The online version contains supplementary material available at 10.1186/s12889-023-16148-y.

## Introduction

Many older adults with low socioeconomic status (SES) are confronted with personal and health problems, while also being forced to live in neighbourhoods characterized by low SES. These neighbourhoods, in which a relatively high number of residents have low SES, also tend to have fewer resources (e.g., shops or facilities), less social participation, and less exchange of informal care [1]. Residents of lower-SES neighbourhoods have a shorter life expectancy than do their counterparts in higher-SES neighbourhoods, even after taking individual SES into account [2, 3]. These results demonstrate the strong effect that a neighbourhood has on health status.

Neighbourhoods can also facilitate the health and well-being of their residents (e.g., through the presence of community buildings that facilitate social participation) [4]. Several studies have demonstrated the importance of social support and social contacts in neighbourhoods as buffers for the disadvantages associated with lower SES, as well as for improving well-being [5, 6]. Because older adults are currently expected to live independently and self-manage their health for as long as possible, social contacts and social support in neighbourhoods could be beneficial in stimulating well-being within this target group. For this reason, health-promotion interventions focusing on improving self-management ability and well-being in older adults might generate better results by embracing the community and its potential for social support.

Community can be defined in several ways [7, 8], including in terms of geographic area (e.g., neighbourhoods, cities, or villages) or in terms of social groups that share a common culture, identity, values, or sense of belonging (e.g., church groups or exercise groups) [7, 9]. In this study, we refer to a community according to a combination of two definitions: 1) as a geographic area (e.g., a street or small village) and 2) as a group of individuals who participate together in activities and share the same interests.

Interventions using a community-based approach focused on changing individual behaviours in the community, as well as on modifying community resources to improve the health of residents [7, 10]. As observed by McLeroy and colleagues [9], three types of community-based approaches can be distinguished: (1) using communities for recruitment, with the intervention focusing primarily on individuals; (2) aiming the intervention at behavioural change through social-ecological processes within the community (e.g., through family, neighbours, or local sports clubs); (3) aiming the intervention at changing the social and/or physical environment (e.g., the availability of resources for healthy eating in the neighbourhood) [7, 9].

As highlighted by review studies, community-based interventions for adults have yielded inconclusive results concerning their effects on health outcomes, largely because of differences in the designs and populations addressed in the various studies [7, 11]. Nevertheless, Brand and colleagues [7] report that community-based interventions were successful in improving healthy eating and physical activity amongst older adults, while reducing fall-risk factors and enhancing physical abilities within this target group [12]. Community-based approaches have yielded more positive effects when interventions are executed in groups or when self-monitoring elements and motivational prompts are added. Moreover, these approaches apparently offer a successful strategy for reaching older adults with low SES [13].

Each of the community-based health-promoting interventions designed for older adults mentioned above focuses exclusively on a single aspect of health (e.g., physical activity or mental health) [12, 13]. In current understandings, however, health is often regarded as entailing more than a purely physical state. For example, Huber and colleagues define health as ‘the ability to adapt and self-manage in the face of physical, mental and social challenges’ [14]. To focus on the overall health of individuals, new community-based interventions for adults with low SES should target the combination of physical, psychological, and social health factors [15], as well as on self-management ability [14].

To our knowledge, no existing health-promoting interventions have targeted these components of health in a single community-based approach for older adults with low SES. We therefore developed a new intervention—‘Community Wise’ (CW): a social-ecological community-based health-promoting intervention executed in low-SES neighbourhoods in the northern and eastern regions of the Netherlands. The intervention was intended to improve the physical fitness, self-management ability, social health, and well-being of older people living in low-SES neighbourhoods.

The main objective of this study is to evaluate the changes in physical fitness, self-management ability, social health, well-being, and perceptions of CW participants, based on a mixed-methods approach. First, we use quantitative measures (i.e., performance tests and questionnaires) to assess whether improvements occurred in physical fitness, self-management ability, social health, and well-being, following a pre-test/post-test experimental design. Second, we draw on qualitative measures (i.e., semi-structured interviews and focus-group sessions) to explore the experiences of participants with regard to the intervention and the resulting improvements.

## Method

### Design and recruitment

The current study was part of a larger project, which was based on a pre-test/post-test experimental design with two follow-up assessments. The pre-test consisted of one baseline assessment (T0), an assessment directly after the intervention finished (T1), and two follow-up assessments: one after 6 months (T2) and the other after 12 months (T3). In the current study, we investigate our research question using a combination of both quantitative and qualitative research methods and measures (performance tests, questionnaires, semi-structured interviews, and focus-group sessions).

Community Wise was developed as a community-based intervention. Participants in the same community or neighbourhood were allocated to the same intervention group. The target group for the intervention consisted of adults living in low-SES communities or neighbourhoods. We initially focused on adults 40 years of age or older. This was because, in the Netherlands groups with lower SES start developing chronic conditions around the age of 40 years, as compared to 49 years for those with higher SES [16]. Because of the community-based approach, however, we did not exclude any community residents who were willing to participate.

Several strategies were used to recruit participants for this study. The first consisted of door-to-door visits. This was done in collaboration with municipalities, social care organizations, and a cooperative building association that identified communities with a relatively high number of older residents with low SES and in which, at that time, no health interventions were offered. We received addresses of inhabitants who were 40 years of age and older within these communities. One week before the door-to-door visits, information letters were distributed in the post boxes of residents, containing information about the upcoming visits, along with contact information, should they not wish to be visited.

Residents were interviewed according to the ‘community scan’, which consisted of a semi-structured questionnaire aimed at obtaining a better understanding of the experiences of residents with their neighbourhoods. It was developed in collaboration with local stakeholders (e.g., social workers or members of the neighbourhood activity committee). The interviews were conducted by trained research assistants and social care (and other) professionals working in the communities. All residents of the community were invited to participate in a health check consisting of a physical fitness test and short conversation with one of the researchers, research assistants or trainers concerning the intervention. During this conversation, the purpose of and programme for the intervention sessions were discussed briefly.

The health check served two purposes. First, it provided a tool for reaching residents and motivating them to participate in the intervention by providing them with information about the intervention. Second it generated a baseline measurement of the physical fitness of participants. The health check was organized for residents in a local community building. During the health check, residents participated in a performance test, received information about the CW intervention, and had the opportunity to enrol in the intervention. Residents who were not at home at time of the door-to-door visits were visited again on a different day. If they were not at home during the second visit, they received written information on whom to contact if they would like to be visited. The second recruitment strategy involved key peers living in the neighbourhood, along with local professionals from home care organizations, social care organizations, and sports organizations. The third strategy consisted of a notice containing information about the CW intervention distributed through social media and local newspapers. The fourth strategy consisted of ‘hallway conversations’, in which the residents of hallways in residential facilities were invited to participate in a group discussion concerning the liveability of the residential facility. After this hallway conversation, residents were invited to participate in the health check and CW intervention.

We performed a power calculation based on the results of the DELFGOUD intervention study (a study of the Groningen Active Ageing Strategy) [17]. The smallest effect size was observed for leg strength (0.21). We therefore performed a power calculation on this outcome measure, with the expectation that the other outcome measures would reach statistical significance with the same sample size. The results (power = 0.8; *p*-value = 0.05; effect size *f* for repeated measures = 0.21) indicated that a total sample of 154 participants. Assuming that about one fourth of the participants would drop out of the study, we aimed to recruit 200 participants.

### The Community Wise (CW) intervention

The CW intervention was based on the theory of Self-Management of Well-being (SMW) [18, 19]. According to this theory, six self-management abilities are important to gaining and maintaining social and physical well-being. The CW intervention comprises a programme of education and exercise, with some exercises from the SMW group intervention [20, 21] and some from the Groningen Active Ageing Strategy (GAAS) programme [22]. More importantly, new exercises were added, which were specifically designed for older adults with low SES. For example, we avoided abstract tasks (prioritizing or clustering) or discussions about abstract subjects, and we included physical exercises suited to the physical abilities of older adults. In addition, the reading and writing exercises of the SMW group intervention were replaced with active group exercises or group discussions.

As a community-based group intervention, CW also aimed to improve social cohesion within the group. The intervention was developed in a team that included the developers of the SMW intervention and the GAAS programme, as well as SMW-certified teachers who also worked with the target group. The SMW group intervention [in Dutch: *GRIP&GLANS groepscursus*] was found to have significant effects in improving self-management ability and well-being, while reducing loneliness [20, 21, 23]. The Groningen Active Ageing Strategy (GAAS) [in Dutch: *Sociaal Vitaal*], which is based on the programme of the Groningen Active Living Model, significantly improved the physical and social vitality of older adults with lower SES through physical exercises and group activities [22, 24, 25].

The CW intervention consisted of 12 weekly sessions, each lasting 90 min. All sessions followed the same structure, starting with a warm-up, followed by one or more exercises aimed at improving various aspects of health, and then drinking coffee or tea with the group and discussing the highlights of the session. Nine of the intervention sessions focused on the combined improvement of physical fitness, self-management ability, social health, and well-being. These sessions included educational exercises, group discussions, and physical activities. The other three sessions were ‘movement classes’, with a focus on physical activities suited to the needs and wishes of the participants. One of the trainers for these sessions was professionally trained to develop sport exercises specifically for older adults. The physical capacities of the participants were taken into account, based on the results of the physical-fitness test. The exercise programme was aimed at enhancing the participants’ muscle strength, endurance, and coordination. For example, low-impact exercises (e.g., chair yoga) were applied in intervention groups consisting primarily of vulnerable older adults. Other groups participated in higher-impact exercises, such as a circuit of multiple physical exercises for older adults. The assessments were conducted during the first and last sessions of the intervention. The final session consisted of a short wrap-up activity and a joint closing, followed by the post-assessment (Details about the programme of the intervention sessions have been described in Dutch [26]).

All CW sessions were guided by two out of a pool of eight qualified trainers, all of whom had experience working with the target group. At least one of the two trainers in each session had been officially trained and certified by the SMW Programme as a SMW group-intervention instructor. Some were also sports trainers for older adults. Prior to the start of the intervention, all eight trainers received the CW manual containing instructions about the purpose and execution of the sessions, as well as a brief training about the CW intervention programme (given by one of the researchers).

### Assessments

The baseline assessment was conducted at the end of the first intervention session. During the break, participants were invited to complete two questionnaires and asked to participate in performance tests. During the final session, participants were asked to complete the same two questionnaires that had been used during the first session and to participate in the performance tests. Each participant was also invited to evaluate the intervention in an individual semi-structured interview and one focus-group session.

### Quantitative data collection

The quantitative data collection was performed by the first author (FP) and a pool of research assistants who had received a brief training course on the physical-fitness test and information about the questionnaires before the start of the data collection**.**

#### Physical fitness

Health-related fitness was measured according to five validated, standardized, performance-based tests. Reliability and validity indicators for the fitness test applied in this study ranged between 0.79 and 0.97. [27]. Aerobic endurance was assessed according to the Two-Minute Step Test [27]. During this test, the participant is asked to march in place while raising the knees as many times as possible for two minutes. Leg strength was assessed according to the 30-s Sit-to-Stand test [27]. In this test, the number of times the participant completed a sitting-to-standing movement without using them arms during a period of 30 s. Dynamic balance was assessed according to the timed ‘Up-and-Go’ test [28], which measures the time that a participant needs to rise from a chair, walk to a cone, and return to the seat. The best score out of two trials was recorded. Grip strength was measured by having the participant squeeze a dynamometer three times with one hand. The best score out of three was documented. Shoulder flexibility was measured by having a participant place one hand on the shoulder and reach around behind the back with the other hand, while trying to touch the fingers of both hands together. If a participant could not touch both hands together, the distance between the hands (in centimetres) was documented.

#### Self-management ability

Self-management ability was measured according to the 30-item Self-Management Ability Scale (SMAS-30), which consists of 30 items and six sub-scales [29]. Each sub-scale refers to one of the six self-management abilities, as elaborated by SMW theory [19]. The sub-scales are scored on five-point or six-point Likert scales and transformed into a 100-point Likert scale. Total scores ranged from 0 to 100, with higher scores indicating greater self-management ability. The internal consistency of the overall scale was 0.86 at T0, and 0.87 at T1.

#### Loneliness

Loneliness was measured according to the six-item Loneliness Scale (i.e., the short version) [30]. Each question had five response options: ‘yes!’, ‘yes’, ‘more or less’, ‘no’, and ‘no!’. As suggested by the authors, the scores were dichotomized, such that the total score ranged from 0 (not lonely) to 6 (extremely lonely) [30]. The internal consistency of this scale was 0.83 at T0 and 0.59 at T1.

#### Social cohesion

Social cohesion in the neighbourhood was assessed according to the eight-item instrument developed by Fone and colleagues [31]. Each question had a five-point response scale ranging from ‘strongly disagree’ to ‘strongly agree’. The internal consistency of this instrument in the current study was 0.82 at T0 and 0.80 at T1.

#### Secondary outcome

#### Well-being

Well-being was assessed according to the short version of the Social Production Function Index Level (SPF-IL) scale [32]. This scale consists of 15 items, with five sub-scales: comfort, stimulation, affection, behavioural confirmation, and status. Each sub-scale contains three items and a total score. All sub-scales are scored on a four-point Likert scale ranging from ‘never’ to ‘always’. The internal consistency of the scale was 0.74 at T0 and 0.79 at T1.

#### Demographic characteristics

We asked participants to indicate their age, gender, marital status, country of origin, education level, and financial situation in a short questionnaire.

#### Quantitative analytical strategy

The data were analysed using IBM SPSS Statistics for Windows, version 26. We executed an independent-sample *t*-test and Chi-squared test to compare the participants who dropped out to those who completed the intervention. To examine possible differences in the outcome variables of physical fitness, self-management ability, social health, and well-being at T0 and T1, we performed a paired-sample *t*-test, with significance defined at a value of *p* < 0.05.

### Qualitative data collection

#### Individual semi-structured interviews

During the final (12^th^) session of the intervention, participants were invited to be interviewed about their experiences with the intervention and their perceptions of improvement in terms of physical fitness, self-management ability, social health, and well-being. The topic guide for the interview was semi-structured and developed in collaboration with the research team. Questions in the interview addressed a variety of topics, including whether participants felt that their physical fitness had improved and how they had experienced the group exercises. The individual interviews were conducted either by one of the researchers or by a trained research assistant.

#### Focus-group sessions and individual interviews

During the final session of the intervention, one of the researchers also held a focus-group session with all participants in the intervention group. A total of 10 focus-group sessions were held. The topics addressed in these discussions differed from those addressed in the individual interviews. More specifically, the focus of the interviews was on individual experiences with the intervention, while the focus-group sessions concentrated on group interactions. For example, during the interview, we discussed the improvements that participants perceived in terms of physical fitness or mental health. During the focus-group discussions, we often focused on the feeling of social connection between group members. There were no additional inclusion criteria for participation. All participants were invited for the group discussion, and they were if they would be willing to participate in a one-to-one conversation for the interview. The focus-group sessions were intended to stimulate a discussion between participants, which could result in a broader understanding of the experiences and perceptions of the participants. The topic guide for the focus-group sessions was semi-structured and developed in collaboration with the research team. Examples of questions included how the group had experienced the intervention programme and the connection that they felt with other participants in the group. The sessions were conducted by the first author (FP), who has been trained to guide focus-group sessions.

#### Qualitative analytical strategy

Throughout the entire analysis, we took measures to enhance the reliability of our results [33]. Both the interviews and focus-group sessions were transcribed verbatim, except for the names and residences of the participants which were substituted with functional codes to ensure the participants’ confidentiality. The transcripts were analysed according to the standards of thematic analysis. This analytic method was selected because it focuses on collective shared meanings and experiences, instead of on unique experiences with a single item [34].

We coded the data in ATLAS.ti version 9 using a combined inductive and deductive approach in the codebook. This approach is well-suited to this study, given its clear and applied focus [35]. After becoming familiar with the data, we developed and defined codes, which were refined as data analysis progressed. The deductive codes followed our research aim, and inductive codes were developed throughout the analyses to ensure a rich description of the data. The codebook included codes on physical health, social health, self-management ability, well-being, and the continuity of the group. Coding was carried out systematically by three research assistants and the first author (FP). To reach consensus about the meaning of the codes, differences in coding were discussed in several meetings with all research assistants and the first author (FP) [36]. After coding all text fragments, codes with the same meaning were merged and codes were clustered into themes. To enhance the transparency of the analytical process, the codes were documented in the codebook, along with all changes to the codes throughout the process and the relationship between codes and themes [37, 38]. The themes capture important aspects in the data in relation to the research question. They were clustered according to similarities and overlaps in the codes [35]. The themes, were used to identify and describe shared meanings and experiences of the participants.

## Results

### Quantitative results

#### Overview of participants

**Fig. 1:**
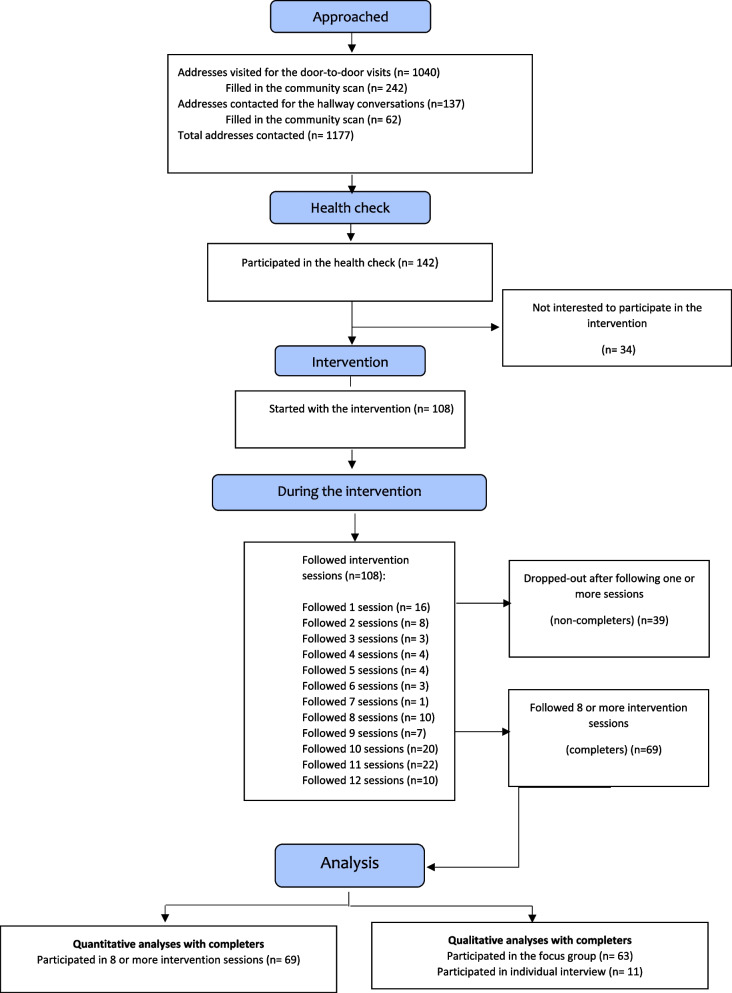
CONSORT Flowchart for participants

The CONSORT flowchart (Fig. 1) depicts the flow of participants through the study. Of the total 1,117 individuals approached in the recruitment process, 142 took a physical fitness test (recruitment rate of 12.7%). Of these participants, 34 participants chose not to start the intervention (due to lack of interest). The mean age of the participants who did not start the intervention was 74 years (*SD* = 16.5), and most (*n* = 23) were female. In all, 108 participants started the intervention and participated in at least one session. After having followed one or more sessions, 39 participants dropped out. We refer to this group as ‘non-completers’. Reasons for dropping out included illness, lack of motivation because the intervention did not meet the participants’ preferences, mobility problems, personal problems, and an argument that occurred within the group. For the analysis of differences in the outcome variables from T0 to T1, we included only the data on participants who followed at least eight sessions (*n* = 69), as this ensured that they had been exposed to activities aimed at all intended elements (physical fitness, self-management ability, social health, and well-being) at least once. We refer to this group as ‘completers’. The characteristics of completers and non-completers are presented in Tables 1 and 2. Significant differences between completers and non-completers at T0 were identified for country of origin, education level, income, and age (see Tables 1 and 2).

**Table 1:** Demographic characteristics of non-completers and completers

	Non-completers (T0) (*n* = 39)		Completers (T0) (*n* = 69)		Difference between completers and non-completers (Chi-squared)
	**%**	**n**	**%**	**n**	***p*-value**
**Gender**					.483
Female	84.6	33	75.4	52	
Male	15.4	6	24.6	17	
**Marital status**					.548
Married	10.3	2	37.7	26	
Divorced	2.6	1	10.1	7	
Widowed	15.4	6	36.2	25	
Unmarried	5.1	2	2.9	2	
Cohabiting	0	0	1.4	1	
Missing	66.7	26	11.6	8	
**Income**					.027*
I can easily make ends meet	12.8	5	53.6	37	
I can exactly make ends meet	20.5	8	31.9	22	
I have difficulty making ends meet, and I sometimes do not make payments on time	2.6	1	0	0	
Missing	64.1	25	14.5	10	
**Education**					.030*
Less than six years of primary school	2.6	1	4.3	3	
Six years of primary school	0	0	4.3	3	
More than primary school, without completing further education	5.1	2	8.7	6	
Trade school	10.3	4	15.9	11	
Secondary vocational education	7.7	3	29	20	
Higher general or pre-university education	0	0	8.7	6	
University/Higher education	0	0	8.7	6	
Other	7.7	3	4.3	3	
Missing	66.7	26	15.9	11	
**Country of origin**					.029*
Netherlands	23.1	9	85.5	59	
Other	7.7	3	4.4	3	
Missing	69.2	27	10.1	7	

% percentage, *n* sub-sample size

^*^ *p* < 0.05 significant

**Table 2:** Results on outcome variables for non-completers and completers

	Non-completers (T0) (*n* = 39)			Completers (T0) (*n* = 69)			Difference between completers and non-completers (*t*-test)
	**M**	**SD**	**n**	**M**	**SD**	**n**	***p*-value**
Age	67	16.1	39	75	10	68	.029*
Leg strength	12.7	4.75	34	13.4	5.94	62	.459
Aerobic endurance	65.6	36.3	30	72	38.4	60	.385
Shoulder flexibility	-4.4	16.1	35	-9.1	16.6	62	.452
Dynamic balance	8.0	4.0	30	8.3	5.0	62	.985
Grip strength	25.6	9.1	36	27.1	8.7	66	.592
Self-management ability	57.0	17.8	10	62.3	11.9	56	.077
Well-being	25.6	6.5	6	26.5	5.2	44	.150
Loneliness	.83	1.7	12	.46	.93	56	.316
Social cohesion	27.0	6.6	11	27.5	4.9	57	.656

*T0* baseline measurement, *M* mean, *SD* standard deviation, *n* sub-sample size

^*^ *p* < 0.05 significant

#### T0–T1 differences in physical fitness, self-management ability, social health, and well-being

The results indicate that the aerobic endurance and shoulder flexibility of the participants improved significantly between from T0 and T1. No significant improvements were found for other aspects of physical health (see Table 3). The self-management ability (and specific multifunctionality of resources) of completers decreased significantly between T0 and T1. No significant differences in social health (loneliness and social cohesion) or well-being were observed between T0 and T1 (see Table 3).

**Table 3:** Paired-sample t-test results of the pre-test/post-test intervention measurement on outcome variables

Outcome variables		M	*SD*	*n*	*t*	df	*p*-value
**Physical Health**							
**Aerobic endurance**	T0	77.01	38.54	46	-2.319	45	.025*
	T1	88.93	33.92	46			
**Leg strength**	T0	13.64	5.71	53	.069	52	.945
	T1	13.60	4.94	53			
**Dynamic balance**	T0	7.55	4.76	50	.301	49	.765
	T1	7.43	3.65	50			
**Grip strength**	T0	27.20	8.74	56	.671	55	.505
	T1	26.68	7.90	56			
**Shoulder flexibility**	T0	-8.23	16.40	51	-2.105	50	.040*
	T1	-1.65	15.99	51			
**Self-management ability**	T0	63.46	12.07	41	2.427	40	.020*
	T1	61.37	10.55	41			
**Self-efficacy beliefs**	T0	73.60	12.69	50	.368	49	.714
	T1	74.12	9.95				
**A positive frame of mind**	T0	66.45	13.36	48	-.335	47	.724
	T1	66.97	11.92				
**Taking the initiative**	T0	56.46	14.67	50	-1.529	49	.133
	T1	56.48	13.61				
**Investment behaviour**	T0	66.66	14.55	48	.706	47	.484
	T1	65.54	13.69				
**Multifunctionality of resources**	T0	53.34	16.50	44	9.195	43	< .001*
	T1	37.54	15.43				
**Variety of resources**	T0	59.56	16.91	48	.557	47	.580
	T1	58.54	14.63				
**Well-being**	T0	26.14	8.19	27	-1.490	26	.148
	T1	27.48	6.53	27			
**Social health**							
**Social cohesion**	T0	28.25	4.23	43	1.050	42	.300
	T1	27.69	4.61	43			
**Loneliness**	T0	.56	1.04	39	.784	38	.438
	T1	.41	.88	39			

*T0* baseline measurement, *T1* post-intervention measurement, *M* mean, *SD* standard deviation, *n* sub-sample size, *t* calculated difference represented in units of standard error, *df* degrees of freedom

^*^ *p* < 0.05 significant

#### Qualitative results of interviews and focus-group sessions

Participants’ experiences of the CW intervention and their perceptions of improvement.

In this section, we describe the results according to the following deductive themes: physical health, social health, self-management ability, well-being, and the continuity of the group. Quotations are provided to illustrate the themes. The characteristics of the intervention participants who also participated in the interviews (interviewees) and focus-group sessions are described in Table 4.

**Table 4:** Demographic characteristics of interviewees and focus-group participants

	Interviewees (*N* = 11)		Focus-group participants (*N* = 63)	
	**M**	**SD**	**M**	**SD**
Age (years)	73	5	76	9
	**%**	**N**	**%**	**N**
**Gender**				
Female	72.2	8	74.6	47
Male	27.3	3	25.4	16
**Marital status**				
Married	45.5	5	39.7	25
Divorced	18.2	2	9.5	6
Widowed	27.3	3	41.3	26
Unmarried	0	0	3.2	2
Cohabiting	0	0	1.6	1
Missing	9.1	1	4.8	3
**Income**				
I can easily make ends meet	72.7	8	57.1	36
I can exactly make ends meet	27.3	3	31.7	20
I have difficulty making ends meet, and I sometimes do not make payments on time	0	0	0	0
Missing	0	0	11.1	7
**Education**				
Less than six years of primary school	0	0	1.6	1
Six years of primary school	0	0	4.8	3
More than primary school, without completing further education	0	0	9.5	6
Trade school	9.1	1	19	12
Secondary vocational education	45.5	5	31.7	20
Higher general or pre-university education	18.2	2	9.5	6
University/Higher education	27.3	3	6.3	4
Other	0	0	4.8	3
Missing	0	0	12.7	8
**Country of origin**				
Netherlands	23.1	9	92.1	58
Other	7.7	3	4.7	3
Missing	69.2	27	3.2	2

*SD* standard deviation, *N* sample size, % = percentage

^*^M = mean

#### Participants’ perceptions of changes in physical fitness due to Community Wise

All of the participants interviewed were positive about the CW movement exercises and reported having found these exercises enjoyable. Almost all interviewees described having been physically healthy before the start of the intervention. These participants had been participating in multiple physical activities (e.g., swimming, cycling, walking, fitness, dancing) before starting CW. For this reason, they did not perceive that the movement exercises had improved their physical health. In addition, multiple interviewees stated that the exercises were not intensive or frequent enough to improve their physical fitness.

*I didn’t notice anything different about my own body. I didn’t find it intensive either, but that could also be because I do quite a lot of sports and exercise myself. (Interviewee 6, female)*

These interviewees also explained that physical differences between individuals in the group influenced the intensity of the ‘movement classes’. Some interviewees would have liked for the exercises to have been more intensive.

During the focus-group sessions, participants were given time to reflect on their perceptions concerning the effects of the intervention on their physical fitness. In general, they talked about the positive effect of the physical exercises on the connection between group members, which they described as having improved—especially when a game element was involved.

*When you exercise together or play games together, you get to know each other in a playful way. (Focus-group session 9)*

Similar to the interviewees, most focus-group participants identified the movement classes and exercises as the most enjoyable parts of the intervention. Participants with more physical vulnerabilities also mentioned the group discussions as important and enjoyable. Some participants experienced the combination of physical exercises and group discussions in one session as difficult. They would have preferred to have had only physical exercises or only group discussions.

#### Participants’ perceptions of changes in social health (social contact with group members and outside the intervention group) due to Community Wise

During both the interviews and the focus-group sessions, the conversation focused specifically on social aspects of the intervention. The social aspect was also mentioned as one of the main reasons for continuing with the intervention programme, specifically for interviewees who reported being physically healthy. According to some interviewees, the intervention also contributed to building and maintaining meaningful relationships outside the intervention group, as participation had improved their social skills and encouraged them to become more interested in the feelings and thoughts of their loved ones. They also felt encouraged to contact people they had not seen for a while:

*I noticed it in how I deal with other people. Yeah, before the intervention, I was always introverted. That’s no longer the case. I notice that I am much more outwardly focused. (Interviewee 2, male)*

During the focus-group sessions, most participants mentioned that CW had helped them to build new and meaningful relationships with members of the intervention group by sharing interests and talking about their personal lives. Participants who had experienced feelings of loneliness before the intervention explained that belonging to a new group and having new social contacts had reduced their loneliness.

*I had locked myself up for some time and had very few social contacts. This group is so enriching; the people here are great. (Focus-group session 5)*

Participants nevertheless differed in their need for social contacts. One main reason was that participants who were living with a partner felt less need for social contacts. In addition, some focus-group participants indicated that they simply did not feel the need for much social contact with other people.

*There are always people who don’t like being in a group. I’m not really a person who likes to be in a group. (Focus-group session 5)*

#### Social connection with members of the intervention group

There was a considerable amount of overlap between the results concerning social contacts and social connection between group members, as participants often mentioned social connection as part of their social contacts within the group. Almost all interviewees stated that the social connection between group members had grown as the intervention progressed.

*I feel really bonded with everyone. At one point. they asked about friendship. Well, I say that these are my friends. (Interviewee 3, female)*

Not all interviewees felt strong connections with all group members. Some noted that some group members did not listen to each other, and that this had disturbed the atmosphere within the group. In some groups, interviewees mentioned that some participants always took the lead in the conversations and that other group members felt that they could not interfere. As explained by one interviewee, differences between group members (in this case, in terms of education level) made it difficult to share interests or passions with the group. In some groups, interviewees felt more connection with group members who had similar interests.

*I can’t actually share the activities that I enjoy myself with the others [group members]. Their interests are different. (Interviewee 8, male)*

During the focus-group sessions, almost all participants were positive about other group members and the atmosphere within the group. The exercises that stimulated sharing personal situations facilitated the social connection within the group. The guidance provided by the trainers (e.g., ensuring a sense of safety to share personal stories, humour, and listening to other group members) contributed to a positive atmosphere within the group.

*Yes, that had grown immensely, that connection. I can see that from how people bring certain things up... someone mentions something and then someone reacts to it later or makes a certain joke about it. (Focus-group session 2)*

During one focus-group session, however, some participants did not feel safe sharing their personal stories during the focus-group session. One participant indicated that she did not feel safe sharing her personal experiences in the group, and this had also affected the feeling of connection withing the group.

#### Social support from members of the intervention group

Social support is defined as emotional or practical help experienced from others [39]. Some interviewees did experience emotional support from other group members during the intervention sessions. Interviewees described experiences with support as listening to one another, showing empathy, and giving advice. No interviewees provided any indication that practical help had been a form of support within the intervention group.

*There was certainly a listening ear; we also gave each other a lot of advice. And what you also do is coming back to each other. That you say, ‘Last time, you had this problem. How are you doing now?’ (Interviewee 11, female)*

Some interviewees indicated that they had not experienced any increase in social support. These interviewees noted that they already had sources of social support outside of the intervention group and that they had no need for additional support. As they explained, they did not always feel an urge to share their personal stories/problems during the intervention sessions:

*I do have a lot of people around me with whom I can talk and express myself. And then I might not have much need for something like that (the intervention). (Interviewee 5, female)*

During the focus-group sessions, the social support between group members was also discussed. The results were similar to those of the interviews. Only one focus-group participant reported not having experienced receiving social support from the group.

#### Participants’ perceptions of changes of in self-management ability due to Community Wise

During the intervention sessions, the phrase ‘control over one’s life’ was used to explain to participants the ability to self-manage aspects of their life/health in plain words. In the interviews, participants were asked whether they perceived having more control over their lives after the intervention, as well as whether they perceived being more resilient. Some participants described ‘control over one’s life’ as the ability to say ‘no’ more often:

*Yes, certainly [more control]. I’ve also become a bit less shy about saying ‘no’ and not always giving in. I no longer feel guilty when saying ‘no’. It just feels good. (Interviewee 8, male)*

Some interviewees did not understand the question about resilience and talked about ‘control over one’s life’ and resilience as similar terms. Others apparently understood resilience in physical terms, describing it as the ability to exercise for a long period of time. It is therefore not clear whether participants understood the definition of resilience within context of the intervention.

*I’ve participated in sports for more than 40 years in my life, so, yes, I have resilience. (Interviewee 2, male)*

Although participants did not specifically mention the term ‘self-management ability’, analysis of both the interviews and focus-group sessions revealed several self-management abilities of the participants, based on SBW theory [18]. The most frequently mentioned was *taking initiative*. More specifically, interviewees reported that the intervention had positively affected their tendency to take initiative in their personal lives and to engage in new activities or new social relationships, which they had avoided before the start of the intervention. Some interviewees also started to volunteer in the community after the intervention, which they had previously not dared to do. They noted that support from group members and trainers had helped them to take these steps. Interviewees also described having more self-confidence and trying to have a positive mindset more often. These aspects are part of the self-management abilities *self-efficacy* and *positive frame of mind*.

*At first, I found it difficult to find any positive aspects of myself. Later, when I could think of more positive qualities, I realized, ‘I am good as I am’. (Interviewee 10, female)*

Not all participants improved or talked about their self-management ability. One common feature shared by these participants was that they had other means of maintaining their self-management ability (e.g., through existing social networks). During the focus-group sessions, participants were asked whether they perceived having more resilience and control over their lives. These issues nevertheless received less attention in the focus-group sessions than they did in the interviews. During one focus-group session, participants reported having become more aware about how to self-manage their lives. Other focus-group participants noted that they had learned how to create a positive mindset.

#### Participants’ perceptions on changes in well-being due to Community Wise

As mentioned in the title of the SMW group intervention, the word ‘glow’ (in Dutch, *glans*) can be used to represent well-being in life, while the word ‘grip’ can refer to the ability to self-manage one’s own well-being. As a part of the CW intervention, participants were taught to think about their current situation with regard to the five dimensions of well-being, as specified in SMW theory, by using a ‘well-being plate’. Derived from five basic human physical and social needs, the dimensions of well-being include the need for comfort and stimulation (physical needs), as well as for affection, behavioural confirmation, and status (social needs) [20]. The following question was specifically included in the interview: ‘Do you think that the intervention has helped you give more glow to your life?’ Participants gave a variety of answers, which were similar to their responses concerning resilience and having control over one’s life. Some participants had difficulty answering the question.

*Well, more glow in life…I think it’s such a vague term. I don’t really know. (Interviewee 6, female)*

Participants also varied widely in terms of who did and did not enjoy working with the well-being plate. Many of those who did not like to work with it mentioned a specific preference for the physical exercises of the intervention. According to these participants, working with the well-being plate was more suitable for vulnerable older adults.

*There are a lot of these kind of courses that have the same subject. For a lot of people, that would be ideal. I don’t need it. (Interviewee 8, male)*

Interviewees who enjoyed working with the well-being plate spoke primarily about improving social contacts and learning how to cope with negative emotions. They also reported having become more aware about their personal situations.

During the focus-group sessions, answers also varied widely between intervention groups. Groups with more physically vulnerable older adults enjoyed working with the well-being plate more, as compared to groups of participants with fewer physical vulnerabilities.

#### Continuation of the group after the end of Community Wise

In both the interviews and focus-group sessions, participants were asked if they would like to continue with the group after completing the intervention. In addition, during the 11^th^ intervention session, trainers asked participants to think about the continuation of the group. Although interviewees often expressed interest in continuing the group, the types of activities remained unclear, and many possible activities were suggested. The interviewees specifically highlighted the importance of group continuation to maintaining social connection between group members.

The continuity of the group was discussed in greater depth during the focus-group sessions. In these sessions, most participants expressed a desire to stay in contact with the group. At the same time, however, they noted that group activities should be less frequent than once a week, so that they could also engage in other social (or other) activities. The desired type of group activity also depended on the physical fitness of the group members. Those who were less healthy physically showed more interest in activities that involved talking or playing games.

*Get together once a month, and then play a game and exercise, or discuss things once in a while. (Interviewee 5, female)*

Despite expressing interest in continuing the group, participants found it difficult to make concrete plans. All participants stated that a group leader (professional or member of the group) would be needed if the group were to be continued. None of the participants wanted to do this, however, as it would require too much work. Participants with previous experience or skills in group leadership mentioned that the group leader should be someone younger, given the energy and time needed to organize the group activities.

*We need someone younger with motivation and vitality, someone who can think outside the box. (Focus-group session 1)*

## Discussion

The main objective of this study was to assess pre-test/post-test differences in physical fitness, self-management ability, social health, and well-being. A secondary objective was to evaluate participants’ perceptions of their improvement and experiences with CW, as well as their interest in continuing the group. Although the use of mixed methods gave us a broad understanding of the effects of the intervention, the results of this study are inconclusive. Analysis of the quantitative data revealed that participants experienced only limited physical improvements, and no improvements were found for self-management ability, social health, or well-being. In contrast, the qualitative data analysis indicated that participants experienced improvements in their social health and self-management ability. In this section, we discuss the results for each outcome variable.

### Physical fitness

The quantitative and qualitative data revealed contradictory results. Although the performance tests did reveal some improvements, the interviewees and focus-group participants did not perceive any improvement in their physical fitness. The results of the performance tests showed significant improvements in the aerobic endurance and shoulder flexibility of completers. Given that aerobic endurance is known to decline in older adults over time [40], improvements in the aerobic endurance of participants after following the intervention suggests that the participants did experience some improvement in physical fitness. At the same time, however, the improvements reflected in the performance tests might have been due to measurement errors. More specifically, the performance tests were administered by multiple research assistants. Although all research assistants received instructions, some might not have measured all tests accurately, thus possibly leading to inaccuracies.

According to the qualitative analyses, the interviewees did not experience any improvement in their physical fitness. The difference in results might be explained by the representativeness of the interviewees and their level of physical health before the intervention. These participants volunteered to be interviewed, and all had engaged in many physical activities before starting the intervention. Their experiences might thus have differed from those of the participants who were not interviewed. Given that aerobic fitness decreases in older adults over time (as mentioned before), maintaining the same level of fitness could also indicate that decline was prevented. In addition, within some intervention groups, some participants were more physically vulnerable than others were. Because the trainers needed to take into account the physical level of all participants in the group, the exercises were not intensive enough for those with a higher physical level. All things considered, the physical health of the CW participants was thus either maintained or slightly improved. For future CW intervention groups, it could be advisable to divide the group according to physical level during the movement classes, in order to meet the needs and wishes of all participants.

### Self-management ability and well-being

The quantitative and qualitative data also revealed contradictory results on self-management ability. According to the analysis of the qualitative data, participants perceived that they had improved in terms of having more control over their lives and taking more initiative, in addition to having a more positive frame of mind and increased self-efficacy.

Surprisingly, analysis of the quantitative data revealed a significant decrease in self-management ability, and specifically with regard to multifunctionality of resources. The analysis revealed no differences in well-being. We are convinced that this decrease was not caused by the CW intervention, but by other factors. First, life events (e.g., changes in personal situations, such as the loss of a loved one) might have influenced the self-management ability of participants (e.g., having a positive frame of mind). Second, older adults with lower SES might have more trouble with tasks that require a higher level of literacy (e.g., completing questionnaires) [41]. We noticed that the last part of the questionnaire, which included the questions about self-management ability and well-being, was especially prone to missing data. Given that the data collection was conducted at the end of the intervention session, it is possible that some participants had already become tired from answering some questions and participating in the performance test, and they therefore did not complete the last part of the questionnaire. We also noticed that some participants did not complete the questionnaire in a serious manner. In some groups, participants were talking with each other and joking around during the data collection, thus indicating that these participants were not completely focused on completing the questionnaires.

The qualitative data do indicate that participants showed some improvement in self-management ability. More specifically, participants reported taking more initiative, being more aware of having a positive frame of mind, and exhibiting more self-efficacy. In addition, some participants perceived having more control over their lives. It is possible, however, that the participants did not fully understand the questions related to self-management ability. For example, some participants asked for clarification of the question during the interview, thus indicating that this question might have been too difficult for some participants. Analysis of the data revealed that focus-group participants had used different definitions or had not answered the question about self-management ability. In future CW intervention studies, we recommend adding an explanation of the concepts used in the interview questions, in addition to evaluating the feasibility of the interview questions for the target group in advance of the actual data collection.

### Social health

Our results pointed to a marginal improvement in the social health of CW participants. While the quantitative data analyses did not show any improvement in loneliness or social cohesion, the qualitative data analyses indicated that some participants perceived some improvement in their social skills and social networks. The finding that the social health of completers (in terms of loneliness and social cohesion) showed no significant improvement might be explained by the relatively high level of social health that the participants had prior to the start of the intervention, thus resulting in a ‘ceiling effect’ [42]. According to the qualitative analysis, participants reported experiencing improvement in their social skills, as well as in both the quality and quantity of their social networks. Interviewees who said that they had not experienced any improvement in their social health explained that they already had social networks and that they did not feel the need to invest in new social relationships. This result might also reflect a ‘ceiling effect’ [42]. During the focus-group sessions, almost all participants seemed to agree that their social health had improved. This difference between the interviews and focus-group sessions might have be caused by social desirability bias, as participants in the focus-group sessions might have found it difficult to tell the group that they had not experienced any improvement in their social health or that they felt no need to invest in new social relationships [43].

### Practical implications of using a community-based approach

This intervention study was conducted according to a community-based social-ecological approach. Using this approach, we were able to include 108 participants from a target group that is known for being difficult to reach: older adults with low SES [44]. This relatively low inclusion rate is in line with multiple studies recruiting older adults for health-promotion interventions using a community-based approach [7, 45]. The information that we provide in this study on the characteristics of completers and non-completers of the intervention (Table 1 and 2) could be used to adjust interventions in future studies to make them more suitable for those fitting the description of the non-completers in our study (e.g., non-completers were significantly younger than completers). An intervention programme held in the evening or during the weekend, with a focus on younger older adults, could potentially be more successful in reaching a larger target group.

The sustainability of the intervention was enhanced by its effect on the social networks of participants: eight of the nine intervention groups organized new sessions or activities as a group together after the end of the intervention. These results are in line with those reported by Levasseur and colleagues [4], who suggest that working with local peers can increase the impact of an intervention. The fact that our findings identified only minor physical improvements might have been due to the intensity and duration of the intervention, as suggested by Luten, Reijneveld, Dijkstra, and de Winter [13]. Other studies using a community-based approach for both older adults and individuals with lower SES have reported that community-based approaches have been associated with positive changes in terms of physical activity, dietary fat intake, weight loss, loneliness, and interest in life. One common feature of these studies is that they were based on interventions of high intensity or relatively long duration (e.g., 6 months or one year) [46–48]. Although the SMW group intervention was found to have positive effects on self-management ability, loneliness, and well-being after six weeks [21, 23], future community-based interventions might consider using interventions of greater intensity or longer duration in order to increase the effect on improving physical health.

### Contribution to mixed-method research

The use of mixed methods is known to support the development and evaluation of intervention studies [49]. In this study, we used a combination of quantitative and qualitative research methods to evaluate the effects of the intervention. Although the combination of research methods yielded a broad understanding of the intervention’s effects, the questionnaires used in this study might not have been the most suitable for our target group. In general, however, questionnaires remain useful for assessing pre-test/post-test differences, and possibly for comparing interventions that have been assessed with similar instruments. We therefore recommend pre-testing the concepts measured in questionnaires for future research involving this target group. For example, the questionnaires could be discussed during focus-group sessions with older adults to gain more insight into what they need in order to be able to complete the instruments properly. Similarly, any questions to be used in interviews and focus-group sessions concerning abstract topics should also be pre-tested with the target group. After pre-testing, we are convinced that mixed methods are of value for assessing the effects of intervention.

### Strengths and limitations

This study has several strengths. First, the use of both quantitative and qualitative measurements yielded broad insight into the effects of the CW intervention. The combination of research methods also enhanced understanding of the advantages and disadvantages of using these methods with this target group. Second, the reliability the triangulation built into the research design (i.e., the combination of interviews and focus-group sessions) enhanced the reliability of our findings [33, 37]. Third, instead of focusing on a single component of health, the community-based approach allowed the CW intervention to address multiple aspects, thereby providing a useful first step towards promoting health within this target group, which is known for being difficult to reach.

Our study is also subject to several limitations. First, as a result of the community-based approach, we included some participants who already had higher levels of physical health, self-management ability, social health, and well-being. These participants did not show much improvement after following the intervention. As evidenced in the literature, individuals who are in most need of health interventions are unlikely to participate, and those who do participate usually have higher levels of health [50, 51]. For future CW interventions, we recommend recruiting more vulnerable participants by requesting assistance from local social workers and general practitioners.

A second limitation of our study is that it is based on a pre-test/post-test design without a control group. It is therefore unclear whether the effects of the intervention should be attributed to the intervention alone or whether external factors were also at play. Although we initially intended to include a control group, the number of individuals willing to participate within each community was too small to divide them into an intervention and a control group.

A third limitation has to do with the programme fidelity of the intervention used in the study, which might have caused the inconclusive effects of the intervention. During interviews with the intervention trainers, we learned that some exercises had been adapted to the needs of participants. Moreover, some exercises had been postponed due to circumstances during the intervention sessions. For example, when one participant needed extra attention and time to speak about a personal problem in the group, some trainers found it difficult to start another physical exercise and allowed more time to the group discussion. For future CW interventions, the programme should be further developed and evaluated with assistance from the intervention trainers.

## Conclusion

Community Wise is a community-based health-promotion intervention focused on improving the physical fitness, self-management ability, social health, and well-being of older adults living in communities characterized by lower socioeconomic status. The results of our study are inconclusive, and they suggest that the intervention had only minor positive results on the physical fitness of participants. At the same time, however, the qualitative data indicated that participants perceived that the CW had had positive effects on various aspects of their self-management ability, social health, and well-being. The interviews and focus-group sessions with participants thus apparently generated broader insight into the effects of CW on concepts other than those assessed in the questionnaires. For future research, several aspects of the intervention should be improved, including programme fidelity, specific recruitment of older adults with poor health status, and the tools used to assess the effects of the intervention.

This study was the first step in evaluating the CW intervention. The results should be interpreted in light of the complexity of developing and executing a community-based health-promotion intervention for this target group. We hope that this manuscript will serve as a stepping stone in working towards the development of successful community-based interventions aimed at improving the physical fitness, self-management ability, social health, and well-being of older adults with low SES.

## Supplementary Information

**Additional file 1: S1.** Outline of the intervention sessions.

## Data Availability

The datasets used during the current study are available from the corresponding author upon reasonable request.

## References

[1] Snel E, Custers G, Engbersen G (2018). Ongelijkheid in de Participatiestad. Mens en Maatschappij.

[2] Meijer M, Röhl J, Bloomfield K, Grittner U (2012). Do neighborhoods affect individual mortality? A systematic review and meta-analysis of multilevel studies. Soc Sci Med.

[3] Posadzki P, Pieper D, Bajpai R, Makaruk H, Könsgen N, Neuhaus AL, Semwal M (2020). Exercise/physical activity and health outcomes: an overview of Cochrane systematic reviews. BMC Public Health.

[4] Levasseur M, Généreux M, Bruneau JF, Vanasse A, Chabot É, Beaulac C, Bédard MM (2015). Importance of proximity to resources, social support, transportation and neighborhood security for mobility and social participation in older adults: results from a scoping study. BMC Public Health.

[5] Bowling A, Barber J, Morris R, Ebrahim S (2006). Do perceptions of neighbourhood environment influence health? Baseline findings from a British survey of aging. J Epidemiol Community Health.

[6] Cramm JM, Van Dijk HM, Nieboer AP (2013). The importance of neighborhood social cohesion and social capital for the wellbeing of older adults in the community. Gerontologist.

[7] Brand T, Pischke CR, Steenbock B, Schoenbach J, Poettgen S, Samkange-Zeeb F, Zeeb H (2014). What works in community-based interventions promoting physical activity and healthy eating? A review of reviews. Int J Environ Res Public Health.

[8] MacQueen KM, McLellan E, Metzger DS, Kegeles S, Strauss RP, Scotti R, Blanchard L, Trotter RT (2001). What is community? An evidence-based definition for participatory public health. Am J Public Health.

[9] McLeroy KR, Norton BL, Kegler MC, Burdine JN, Sumaya CV (2003). Community-based interventions. Am J Public Health.

[10] Bruce ML, Smith W, Miranda J, Hoagwood K, Wells KB (2002). Community-based interventions. Ment Health Serv Res.

[11] Wagner EF, Swenson CC, Henggeler SW (2000). Practical and methodological challenges in validating community-based interventions. Children's Serv.

[12] Shumway-Cook A, Silver IF, LeMier M, York S, Cummings P, Koepsell TD (2007). Effectiveness of a community-based multifactorial intervention on falls and fall risk factors in community-living older adults: a randomized, controlled trial. J Gerontol A Biol Sci Med Sci.

[13] Luten KA, Reijneveld SA, Dijkstra A, de Winter AF (2016). Reach and effectiveness of an integrated community-based intervention on physical activity and healthy eating of older adults in a socioeconomically disadvantaged community. Health Educ Res.

[14] Huber M, Knottnerus JA, Green L, Van Der Horst H, Jadad AR, Kromhout D, Smid H (2011). How should we define health?. BMJ.

[15] Montiel C, Radziszewski S, Prilleltensky I, Houle J (2021). Fostering positive communities: a scoping review of community-level positive psychology interventions. Front Psychol.

[16] Knoops K, van den Brakel M (2010). Rijke mensen leven lang en gezond: Inkomensgerelateerde verschillen in de gezonde levensverwachting. TSG.

[17] Bielderman A, de Greef M, van der Schans C (2014). Pleidooi voor een combinatie van krachttraining en leefstijlprogramma’s voor het behoud van zelfredzaamheid van sedentaire ouderen. Jaarboek Fysiotherapie Kinesitherapie.

[18] Steverink N, Lindenberg S, Slaets JP (2005). How to understand and improve older people’s self-management of wellbeing. Eur J Ageing.

[19] Steverink N, Pachana NA, Laidlaw K (2014). Successful development and ageing.

[20] Goedendorp MM, Kuiper D, Reijneveld SA, Sanderman R, Steverink N (2017). Sustaining program effectiveness after implementation: The case of the self-management of well-being group intervention for older adults. Patient Educ Couns.

[21] Kremers IP, Steverink N, Albersnagel FA, Slaets JP (2006). Improved self-management ability and well-being in older women after a short group intervention. Aging Ment Health.

[22] De Jong J, Lemmink KA, Stevens M, de Greef MH, Rispens P, King AC, Mulder T (2006). Six-month effects of the Groningen active living model (GALM) on physical activity, health and fitness outcomes in sedentary and underactive older adults aged 55–65. Patient Educ Couns.

[23] Goedendorp MM, Steverink N (2017). Interventions based on self-management of well-being theory: pooling data to demonstrate mediation and ceiling effects, and to compare formats. Aging Ment Health.

[24] De Jong J, Lemmink KA, King AC, Huisman M, Stevens M (2007). Twelve-month effects of the Groningen active living model (GALM) on physical activity, health and fitness outcomes in sedentary and underactive older adults aged 55–65. Patient Educ Couns.

[25] Stevens M, Bult P, de Greef MH, Lemmink KA, Rispens P (1999). Groningen Active Living Model (GALM): stimulating physical activity in sedentary older adults. Prev Med.

[26] Voornhout, R. (2021). The effect of Community Wise on the mental and social health of elderly with low SES. Retrieved from: https://gmw.studenttheses.ub.rug.nl/id/eprint/27183

[27] Rikli RE, Jones CJ (2013). Development and validation of criterion-referenced clinically relevant fitness standards for maintaining physical independence in later years. Gerontologist.

[28] Podsiadlo D, Richardson S (1991). The timed “Up & Go”: a test of basic functional mobility for frail elderly persons. J Am Geriatr Soc.

[29] Schuurmans H, Steverink N, Frieswijk N, Buunk BP, Slaets JPJ, Lindenberg S (2005). How to measure self-management abilities in older people by self-report: The development of the SMAS-30. Qual Life Res.

[30] De Jong Gierveld J, Van Tilburg TG (1999). Reference standards for the loneliness scale. Tijdschr Gerontol Geriatr.

[31] Fone D, Dunstan F, Lloyd K, Williams G, Watkins J, Palmer S (2007). Does social cohesion modify the association between area income deprivation and mental health? A multilevel analysis. Int J Epidemiol.

[32] Nieboer A, Lindenberg S, Boomsma A, Bruggen ACV (2005). Dimensions of well-being and their measurement: the SPF-IL scale. Soc Indic Res.

[33] Lincoln YS, Guba EG (1989). Ethics: The failure of positivist science. Rev High Educ.

[34] Braun V, Clarke V (2014). What can “thematic analysis” offer health and wellbeing researchers?. Int J Qual Stud Health Well Being.

[35] Braun V, Clarke V (2006). Using thematic analysis in psychology. Qual Res Psychol.

[36] Campbell J, Quincy C, Osserman J, Pedersen O (2013). Coding in-depth semistructured interviews: problems of unitization and intercoder reliability and agreement. Sociol Methods Res.

[37] Rose J, Johnson CW (2020). Contextualizing reliability and validity in qualitative research: toward more rigorous and trustworthy qualitative social science in leisure research. J Leis Res.

[38] Williams M, Moser T (2019). The art of coding and thematic exploration in qualitative research. Int Manage Rev.

[39] Gottlieb BH, Bergen AE (2010). Social support concepts and measures. J Psychosom Res.

[40] Tuna H, Edeer A, Malkoc M, Aksakoglu G (2009). Effect of age and physical activity level on functional fitness in older adults. Eur Rev Aging Phys Act.

[41] Platzer F, Steverink N, Haan M, de Greef M, Goedendorp M (2021). The bigger picture: research strategy for a photo-elicitation study investigating positive health perceptions of older adults with low socioeconomic status. Int J Qual Methods.

[42] Elzen H, Slaets JP, Snijders TA, Steverink N (2007). Evaluation of the chronic disease self-management program (CDSMP) among chronically ill older people in the Netherlands. Soc Sci Med.

[43] Bergen N, Labonté R (2020). “Everything is perfect, and we have no problems”: detecting and limiting social desirability bias in qualitative research. Qual Health Res.

[44] Liljas AE, Walters K, Jovicic A, Iliffe S, Manthorpe J, Goodman C, Kharicha K (2017). Strategies to improve engagement of ‘hard to reach’older people in research on health promotion: a systematic review. BMC Public Health.

[45] IBM Corp. IBM SPSS Statistics for Windows, Version 26.0. Armonk: IBM Corp; 2019.

[46] Jenum AK, Lorentzen CAN, Ommundsen Y (2009). Targeting physical activity in a low socioeconomic status population: observations from the Norwegian “Romsås in Motion” study. Br J Sports Med.

[47] Johnson J, Stewart A, Anna N, Acree M, Flatt J, Max W, Gregorich S (2018). A choir intervention to promote well-being among diverse older adults: the community of voices trial. Innov Aging.

[48] Kramer MK, Vanderwood KK, Arena VC, Miller RG, Meehan R, Eaglehouse YL, Kriska AM (2018). Evaluation of a diabetes prevention program lifestyle intervention in older adults: a randomized controlled study in three senior/community centers of varying socioeconomic status. Diabetes Educ.

[49] Nastasi BK, Hitchcock J, Sarkar S, Burkholder G, Varjas K, Jayasena A (2007). Mixed methods in intervention research: Theory to adaptation. J Mixed Methods Res.

[50] Elzen H, Slaets JP, Snijders TA, Steverink N (2008). Do older patients who refuse to participate in a self-management intervention in the Netherlands differ from older patients who agree to participate?. Aging Clin Exp Res.

[51] McCann J, Ridgers ND, Carver A, Thornton LE, Teychenne M (2013). Effective recruitment and retention strategies in community health programs. Health Promot J Austr.

