# Supplementary material for: Community Wise—effects and participant perceptions of a community- based -positive health intervention for older inhabitants of low SES neighbourhoods: a mixed-methods approach
Source: BMC Public Health. 2023 Jun 27;23:1251. doi: 10.1186/s12889-023-16148-y (PMC10303292; doi:10.1186/s12889-023-16148-y)
Supplement: Supplementary file 1 — Additional file 1: S1. Outline of the intervention sessions. [file 12889_2023_16148_MOESM1_ESM.docx]

| Session | topic | purpose | Exercise (duration 90 minutes in total, of which 1 hour of exercises, and 30 minutes of coffee and after-talking/exercises |
| --- | --- | --- | --- |
| 1. | Getting familiar | -Get to know each-other, the teacher | -Warm-up.  -Scottish ballgame variant (learn names).  - Collect all balls (introduction)  -Get to know the Plate of Well-being.  -Cooling down.  -During coffee, talk about the Plate of Well-Being. |
| 2. | Positive perspective | Practice with positive perspective and self-efficacy | - Warming-up  - Rose-colored glasses exercise.  - lemon exercise  - A series of various physical exercises.  -Drink coffee: talk about experiences with positive thinking and let the participants compliment themselves and learn from successes. |
| 3. | Self-diagnose | How do I look at myself and what are my interests. | -Warming up.  - Mass movement exercise.  - Goals for the future.  - Plate of Well-being.  - Cooling down.  -During coffee, talk about goal setting |
| 4. | Movement class | Working on group interaction and physical activity | - Serie of physical exercises. |
| 5. | Taking initiative | Initiative and self-efficacy | - Warming-up.  - Short series of movement exercises with various game material.  - Develop a movement exercise.  - Cooling down.  -During coffee, talk about experiences with taking initiative and achieving goals. |
| 6 |  | Positive perspective  & strengths | - Warming up.  - Investing in a positive frame of mind  - Practice body posture  - Cooling down  - During coffee: bring something (e.g. photograph) of value for you and share with the group |
| 7. | Combine  & collaborate | Multifunctionality: combining parts of the Plate of Well-being | -Warming up.  - Physical exercises in a team  - Combine parts of the plate of Well-being.  - Cooling down. |
| 8. | Movement class | Working on group interaction and physical activity | - Serie of physical exercises |
| 9. | Investing and building reserves (variety) | What do I want to change  What is my long term perspective | -Warming up.  - Physical exercises  - Exercise about friendship.  - Exercise about negative thoughts  -Cooling Down.  - During coffee: discuss Plate of Well-being. |
| 10. | Working together and promoting cohesion | Group responsibilities, learning to collaborate and communicate | - Warming up.  - Physical exercises  - Exercise to improve group connection.  - Cooling Down.  - Drinking coffee: discussion about good communication |
| 11. | Movement class | Working on group interaction and physical activity | - Serie of physical exercises |
| 12. | Investing | Repetition investing + investing skills in the group | -Warming up.  - Develop a physical exercise  - Exercise to improve working together  - Cooling down.  - Drinking coffee: discuss the continuation of the lessons |
